# Chronic Rheumatic Heart Disease in Older Brazilians: Hospitalizations and In-Hospital Deaths, 2015-2024

**DOI:** 10.7759/cureus.118576

**Published:** 2026-10-25

**Authors:** Julia R Teixeira, Isabelle S Ciquini, Alana R da Costa Rosa, Lorenzo F Benedet, Mateus F Marques, Tuany C Bernardi, Thaís C Esteves-Pereira

**Affiliations:** 1 Medicine, Universidade Anhembi Morumbi, São José dos Campos, BRA; 2 Medicine, Universidade Santo Amaro, São Paulo, BRA; 3 Medicine, Centro Universitário Ingá, Maringá, BRA; 4 Medicine, Universidade do Extremo Sul Catarinense, Criciúma, BRA; 5 Medicine, Centro Universitário São Camilo, São Paulo, BRA; 6 Medicine, Universidade Paranaense, Umuarama, BRA; 7 Epidemiology and Public Health, Diniz Institute, Campina Grande, BRA

**Keywords:** aged, brazil, hospitalisation, hospital mortality, rheumatic heart disease

## Abstract

Introduction and objectives: Chronic rheumatic heart disease remains a cause of morbidity in older adults. This study analyzed hospitalizations and in-hospital deaths associated with the disease among Brazilians aged 60 years or older from 2015 to 2024, examining differences by sex and macroregion and temporal trends.

Methods: This ecological time-series study used data from the Brazilian Unified Health System Hospital Information System and official population estimates. Annual crude hospitalization and in-hospital death rates per 100,000 older adults were calculated by sex and macroregion. Joinpoint regression was used to estimate annual and average annual percent changes.

Results: Hospitalizations increased from 2,632 in 2015 to 3,683 in 2024, while crude hospitalization rates changed from 10.56 to 10.78 per 100,000 older adults. In-hospital deaths increased from 362 to 419, whereas corresponding rates declined from 1.45 to 1.23 per 100,000. Nationally, hospitalization rates among women declined by 7.05% annually during 2015-2020 and increased by 10.69% annually during 2020-2024 (both p < 0.001). Among men, rates declined by 6.83% annually during 2015-2021 (p = 0.004) and increased by 16.77% annually during 2021-2024 (p = 0.008). Over the full period, average national trends were not statistically significant for either outcome in either sex. Regional patterns were heterogeneous. In the Northeast, women showed sustained increases in hospitalization rates (5.08% annually; p = 0.021) and in-hospital death rates (11.88% annually; p < 0.001).

Conclusions: Hospitalization rates declined and subsequently rebounded, while population-based in-hospital death rates and their temporal patterns varied by sex and region. These patterns reflect the joint contribution of hospitalization frequency and the proportion of admissions ending in death and should not be interpreted as deaths causally attributable to chronic rheumatic heart disease. Regional variation indicates an uneven distribution of recorded hospital utilization and outcomes but does not, by itself, establish inequalities in access to or quality of care. The recent increase in hospitalizations indicates greater hospital utilization and should not be interpreted as evidence of increased disease incidence. Continued surveillance incorporating both hospitalization rates and the proportion of admissions resulting in death is warranted to better characterize temporal and regional patterns among older adults.

## Introduction

Chronic rheumatic heart disease (CRHD) is the principal cardiovascular sequela of acute rheumatic fever, which results from an immune-mediated response triggered by an oropharyngeal infection with group A *Streptococcus pyogenes*. Recurrent or persistent cardiac involvement can cause inflammation and progressive remodeling of the heart valves, leading to fibrosis, thickening, and permanent valvular deformities. As the disease progresses, these changes may result in heart failure, arrhythmias, thromboembolic events, pulmonary hypertension, and increased mortality [1].

Progression to CRHD is associated with initial and recurrent episodes of rheumatic fever, particularly in the absence of timely diagnosis and treatment and adequate secondary prophylaxis. Despite substantial reductions in its burden in high-income countries, rheumatic heart disease remains a major cause of cardiovascular morbidity and mortality in low- and middle-income countries [2]. Its distribution is strongly associated with social determinants, including poverty, inadequate housing conditions, and limited access to health care. These factors influence both exposure to streptococcal infections and the diagnosis, treatment, and follow-up care of individuals with rheumatic fever and its sequelae [1].

In Brazil, rheumatic heart disease remains an important health concern despite a decline in its burden over recent decades. National data indicate an ongoing burden of disease-related hospitalizations and mortality, as well as a continued need for valvular interventions, with substantial differences by age and geographic region. CRHD in older adults is of particular clinical importance because it may represent the late manifestation of valvular lesions acquired decades earlier, in a population in which cardiovascular disease often coexists with other age-related conditions [3]. In advanced stages, the consequences of valvular disease may include heart failure, arrhythmias, pulmonary hypertension, and thromboembolic events, as well as the need for valve repair or replacement [1].

In this context, examining the hospital burden of CRHD among older adults is particularly relevant amid population aging in Brazil. Characterizing differences by sex and macroregion may inform health service planning and identify priorities for the care of older individuals living with chronic rheumatic valvular disease.

## Materials and methods

Study design

This observational epidemiological study used an ecological time-series design and a quantitative approach based on secondary data. Hospitalizations and in-hospital deaths associated with CRHD among individuals aged 60 years or older in Brazil from 2015 to 2024 were analyzed. Analyses were conducted for Brazil as a whole and its five macroregions (North, Northeast, Southeast, South, and Central-West) and stratified by sex.

Study population and data sources

The study included hospitalization records for CRHD among individuals aged ≥60 years from 2015 to 2024, obtained from the Hospital Information System of the Brazilian Unified Health System (SIH/SUS), available through the Unified Health System Department of Informatics (DATASUS) [4]. CRHD was defined using codes I05-I09 of the International Statistical Classification of Diseases and Related Health Problems, 10th Revision (ICD-10). The numbers of hospitalizations and deaths occurring during these hospitalizations were obtained for each year, sex, and macroregion. Population denominators comprised the total and sex-specific resident populations aged 60 years or older in each geographical unit, obtained from population estimates and projections produced by the Brazilian Institute of Geography and Statistics (IBGE) [5].

Hospitalization and in-hospital death rates

Annual crude hospitalization rates for CRHD were calculated separately for men and women by dividing the number of hospitalizations among individuals aged 60 years or older of each sex by the corresponding resident population of the same sex and age group, in the same year and geographical unit, and multiplying the result by 100,000. Similarly, annual crude in-hospital death rates were calculated for each sex by dividing the number of deaths occurring during hospitalizations for CRHD among individuals aged 60 years or older by the resident population of the same sex and age group, in the corresponding year and geographical unit, and multiplying the result by 100,000.

Temporal trend analysis

Temporal trend analyses were performed using the Joinpoint Regression Program, version 6.1.0 (National Cancer Institute, Bethesda, Maryland) [6]. Separate models were fitted for hospitalization and in-hospital death rates, stratified by sex and macroregion, with each time series comprising 10 annual observations from 2015 to 2024. Continuous piecewise log-linear models were fitted, with calendar year as the explanatory variable and the natural logarithm of the annual population-based rate as the response variable. The minimum number of joinpoints was set at zero and the maximum at two. Model constraints required at least two observations between each series endpoint and the nearest joinpoint, excluding the joinpoint itself, and at least two observations between consecutive joinpoints, excluding both joinpoints. The final model was selected using the weighted Bayesian information criterion (WBIC).

For each segment identified by the selected model, the annual percent change (APC) was calculated as 100 × (exp(β) − 1), where β represents the estimated slope on the natural logarithmic scale. The average annual percent change (AAPC) was calculated to summarize the overall trend across the 2015-2024 study period, using the duration-weighted average of the segment-specific slopes. For models without joinpoints, the AAPC was numerically identical to the APC. Estimates were reported with their corresponding 95% confidence intervals, calculated using the empirical quantile method. Statistical significance was assessed using a two-sided significance level of 5% (p < 0.05). Given the limited number of annual observations, the number and location of joinpoints were constrained to reduce the risk of overfitting and the identification of segments supported by too few observations. The resulting APC estimates were interpreted in light of the limited temporal resolution of the data. The evaluation of error variance assumptions and temporal autocorrelation was not specified in the analytical protocol.

Ethical aspects

The ethical principles established by Resolution No. 466 of December 12, 2012, of the National Health Council were observed. As the study relied exclusively on secondary, aggregated, anonymized, and publicly accessible data, with no possibility of individual identification, it was exempt from review by a Research Ethics Committee.

## Results

Hospitalizations

From 2015 through 2024, 27,046 hospitalization events for CRHD were recorded among adults aged 60 years or older in Brazil, with a mean annual count of 2,704.6 (standard deviation [SD], 508.1). Annual hospitalizations increased from 2,632 to 3,683 (+39.93%), while the crude hospitalization rate increased from 10.56 to 10.78 per 100,000 older adults (+2.04%). Both measures reached their lowest levels in 2020, at 2,023 hospitalizations and 6.75 per 100,000, before increasing through 2024.

Women accounted for 13,816 hospitalizations (51.08%) and men for 13,230 (48.92%). The female share increased from 49.70% in 2015 to 52.32% in 2024. Sex-specific hospitalization rates were consistently higher in men. From 2015 to 2024, rates changed from 11.94 to 11.65 per 100,000 older men and from 9.46 to 10.09 per 100,000 older women.

The Southeast accounted for the largest share of hospitalizations over the study period, with 11,900 events (44.00%), followed by the Northeast (6,282; 23.23%), South (5,256; 19.43%), Central-West (2,524; 9.33%), and North (1,084; 4.01%). In 2024, crude hospitalization rates were highest in the Central-West (12.61 per 100,000 older adults), followed by the Northeast (12.35), South (11.81), Southeast (9.70), and North (7.58).

The Northeast was the only region with a higher crude hospitalization rate in 2024 than in 2015, increasing from 8.13 to 12.35 per 100,000 older adults (+51.88%). Hospitalizations in this region more than doubled, from 519 to 1,046 (+101.54%). Rates declined between these two years in the remaining regions, with the largest relative reduction in the Central-West, from 18.93 to 12.61 per 100,000 (−33.39%).

Joinpoint regression identified a decline followed by an increase in national hospitalization rates in both sexes. Among women, rates decreased from 2015 to 2020 (APC = −7.05%; p < 0.001) and increased from 2020 to 2024 (APC = 10.69%; p < 0.001). Among men, the reversal occurred in 2021, with a significant decline during 2015-2021 (APC = −6.83%; p = 0.004) followed by a significant increase during 2021-2024 (APC = 16.77%; p = 0.008). The overall AAPC was 0.45% in both sexes and was not statistically significant in either women (p = 0.637) or men (p = 0.811).

Among women, significant declines followed by increases were observed in the North, Southeast, and South. In the North, the APC changed from −15.42% during 2015-2020 to 18.31% during 2020-2024. Corresponding estimates were −5.37% during 2015-2021 and 15.47% during 2021-2024 in the Southeast, and −8.96% and 11.74% over the same intervals in the South. The South nevertheless showed a significant overall decline (AAPC = −2.53%; p = 0.005). The Northeast was the only region without a joinpoint among women, showing a sustained increase throughout 2015-2024 (APC = 5.08%; p = 0.021). In the Central-West, neither the decline through 2020 nor the subsequent increase was statistically significant (Figure 1).

**Figure 1:**
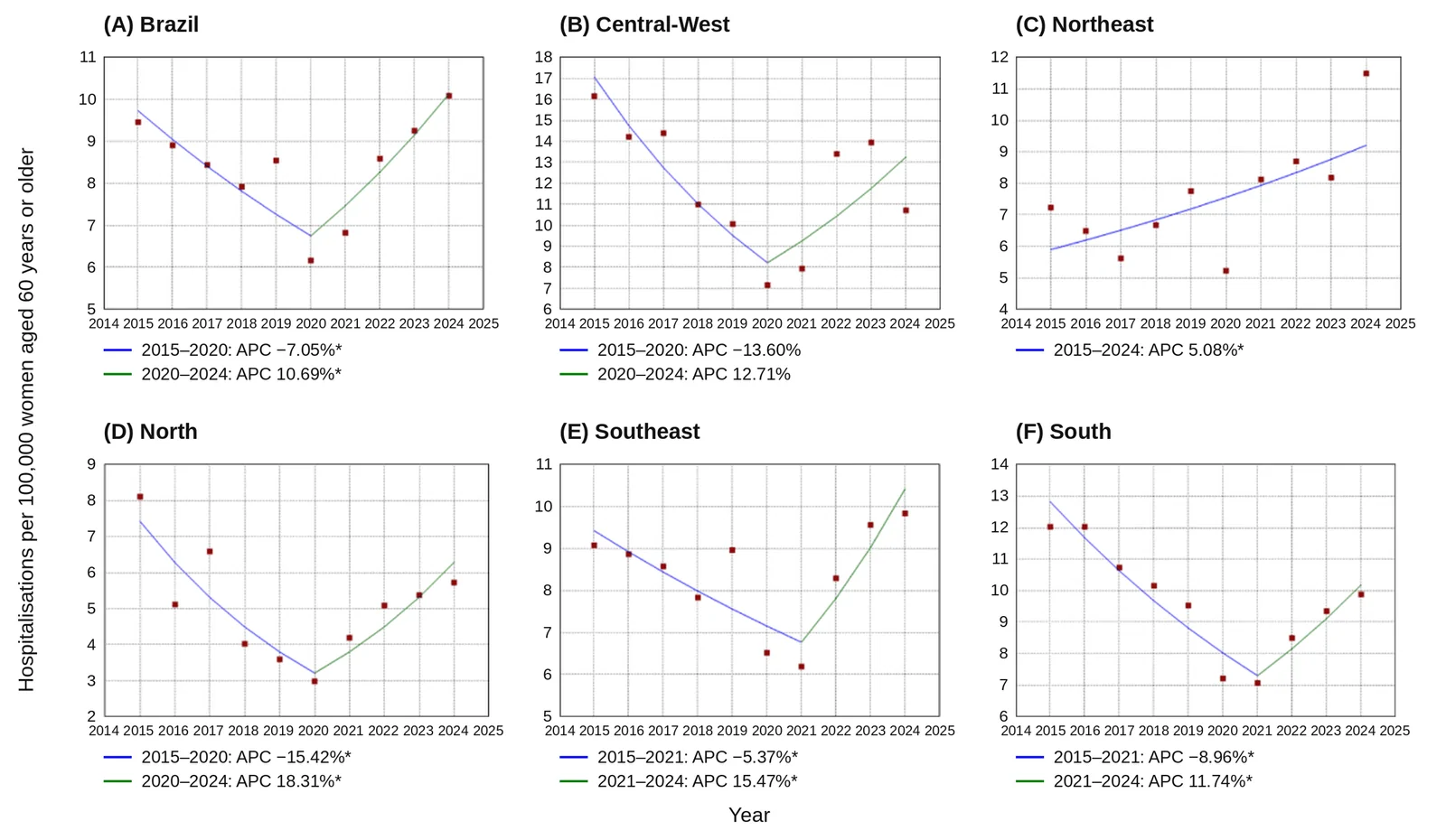
Temporal trends in hospitalization rates for CRHD among women aged 60 years or older in Brazil and its macroregions, 2015–2024. Authors’ analysis of SIH/SUS–DATASUS data using Joinpoint Regression Program v6.0.1 (National Cancer Institute, Bethesda, Maryland).  Panels: (A) Brazil; (B) Central-West; (C) Northeast; (D) North; (E) Southeast; (F) South. One joinpoint was selected in (A), (B), and (D–F); none was selected in (C). * Annual percent change significantly different from zero at the 0.05 level. Blue line, first fitted segment; green line, second fitted segment, where present; red squares, observed annual rates. CRHD: chronic rheumatic heart disease; SIH/SUS: Hospital Information System of the Unified Health System; DATASUS: Department of Informatics of the Unified Health System; APC: annual percent change.

Among men, the North showed a pronounced reversal, with a significant decline during 2015-2021 (APC = −14.94%) followed by a significant increase during 2021-2024 (APC = 30.56%). The Southeast followed a similar pattern, with APCs of −7.90% before 2021 and 14.02% thereafter, both statistically significant. In the Central-West, the decline through 2020 was significant (APC = −16.19%), whereas the subsequent increase was not (APC = 17.58%; p = 0.052). The Northeast showed no significant trend during 2015-2020, followed by a significant increase during 2020-2024 (APC = 14.04%), yielding a positive overall trend (AAPC = 4.15%; p < 0.001). Neither segment was statistically significant in the South (Figure 2).

**Figure 2:**
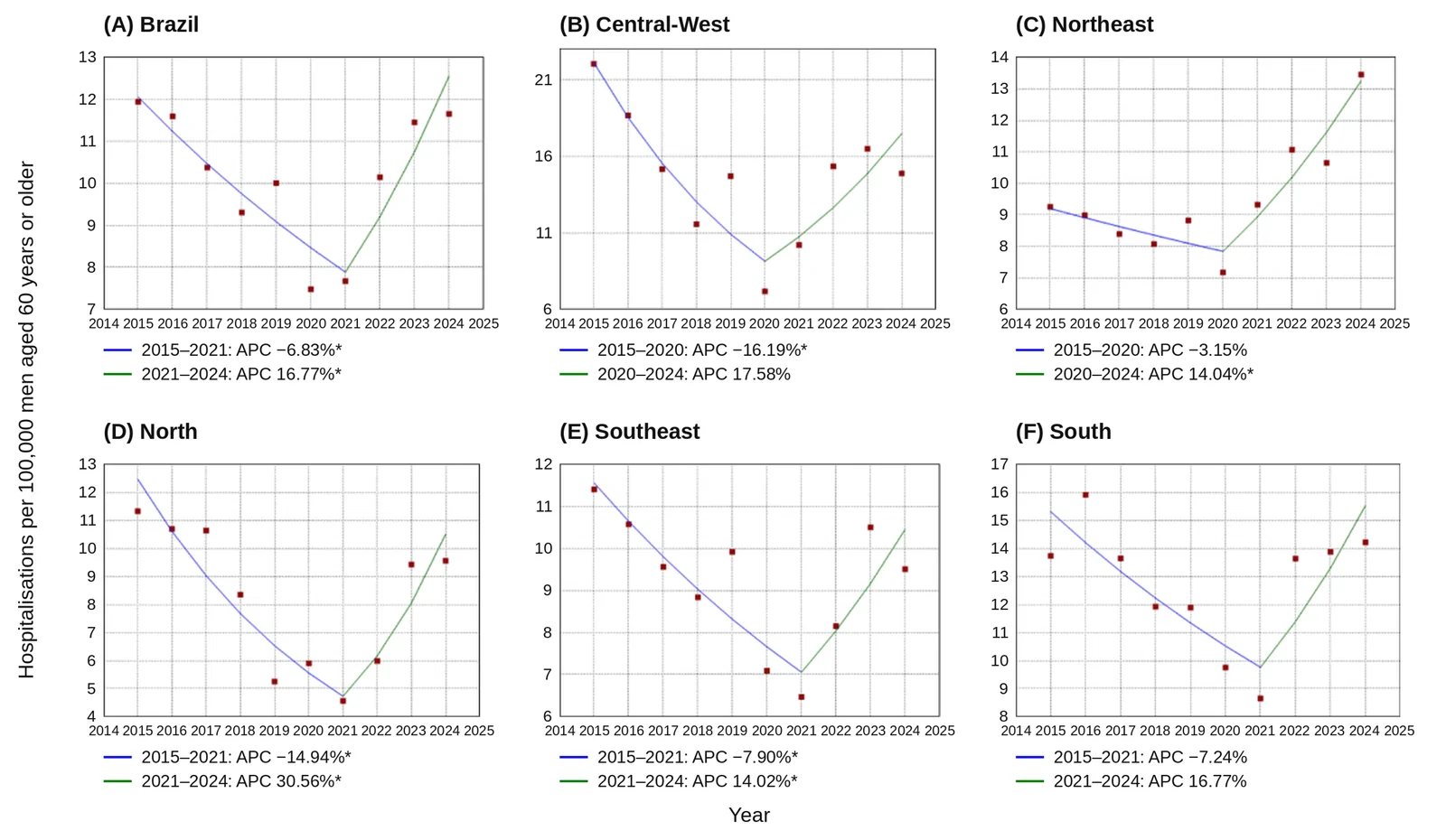
Temporal trends in hospitalization rates for CRHD among men aged 60 years or older in Brazil and its macroregions, 2015–2024. Authors’ analysis of SIH/SUS–DATASUS data using Joinpoint Regression Program v6.0.1 (National Cancer Institute, Bethesda, Maryland). Panels: (A) Brazil; (B) Central-West; (C) Northeast; (D) North; (E) Southeast; (F) South. One joinpoint was selected in every panel. *Annual percent change significantly different from zero at the 0.05 level. Blue line, first fitted segment; green line, second fitted segment, where present; red squares, observed annual rates. CRHD: chronic rheumatic heart disease; SIH/SUS: Hospital Information System of the Unified Health System; DATASUS: Department of Informatics of the Unified Health System; APC: annual percent change.

In-hospital deaths

During the study period, 3,314 deaths occurred during hospitalizations for CRHD among older adults in Brazil, with a mean annual count of 331.4 (SD, 50.0). Annual deaths increased from 362 in 2015 to 419 in 2024 (+15.75%). The number of deaths reached its lowest level in 2020, with 267 deaths. The crude in-hospital death rate decreased from 1.45 per 100,000 older adults in 2015 to 1.23 per 100,000 in 2024 (−15.59%). However, the year of the lowest observed national rate requires confirmation against the annual population denominators and sex-specific data, as the sex-stratified figures show different temporal patterns.

Women accounted for 1,894 deaths (57.15%), compared with 1,420 among men (42.85%). Women represented the majority of deaths throughout the study period, with their share increasing from 52.76% in 2015 to 59.19% in 2024. Between 2015 and 2024, the sex-specific in-hospital death rate decreased from 1.54 to 1.13 per 100,000 older men and from 1.38 to 1.30 per 100,000 older women. The annual trajectories differed between the sexes, as illustrated in Figures 3, 4, with the female rate reaching a visibly lower observed value in 2021 and the male rate remaining low in both 2020 and 2021.

The Southeast accounted for the largest cumulative number of deaths (1,447; 43.66%), followed by the Northeast (694; 20.94%), South (681; 20.55%), Central-West (339; 10.23%), and North (153; 4.62%). In 2024, the crude in-hospital death rate was highest in the South (1.65 per 100,000 older adults), followed by the North (1.41), Northeast (1.17), Southeast (1.10), and Central-West (1.07). The Northeast was the only region with a higher rate in 2024 than in 2015, increasing from 0.94 to 1.17 per 100,000 (+24.34%). The largest relative reduction occurred in the Central-West, from 2.99 to 1.07 per 100,000 (−64.21%).

Overall, 12.25% of hospitalization events ended in death. This proportion was higher among women than men (13.71% vs. 10.73%) and ranged across regions from 11.05% in the Northeast to 14.11% in the North. Nationally, the proportion decreased from 13.75% in 2015 to 11.38% in 2024.

Joinpoint analysis showed different temporal patterns by sex. Among women, no joinpoints were identified, and the national in-hospital death rate showed no statistically significant trend over the study period (APC = AAPC = −2.50%; p = 0.506). Among men, rates declined significantly during 2015-2021 (APC = −19.53%; p = 0.016), followed by an increase during 2021-2024 that did not reach statistical significance (APC = 45.20%; p = 0.078). The overall trend in men was also not statistically significant (AAPC = −2.04%; p = 0.486).

Regional trends among women varied substantially. Rates declined throughout the study period in the Central-West (APC = −15.21%; p < 0.001) and increased in the Northeast (APC = 11.88%; p < 0.001). In the Southeast, a significant decline during 2015-2021 (APC = −14.05%) was followed by a nonsignificant increase. In the South, rates decreased significantly during 2015-2022 (APC = −5.36%) and increased significantly during 2022-2024 (APC = 17.05%). Although a joinpoint was identified in the North in 2022, neither segment showed a statistically significant trend (Figure 3).

**Figure 3:**
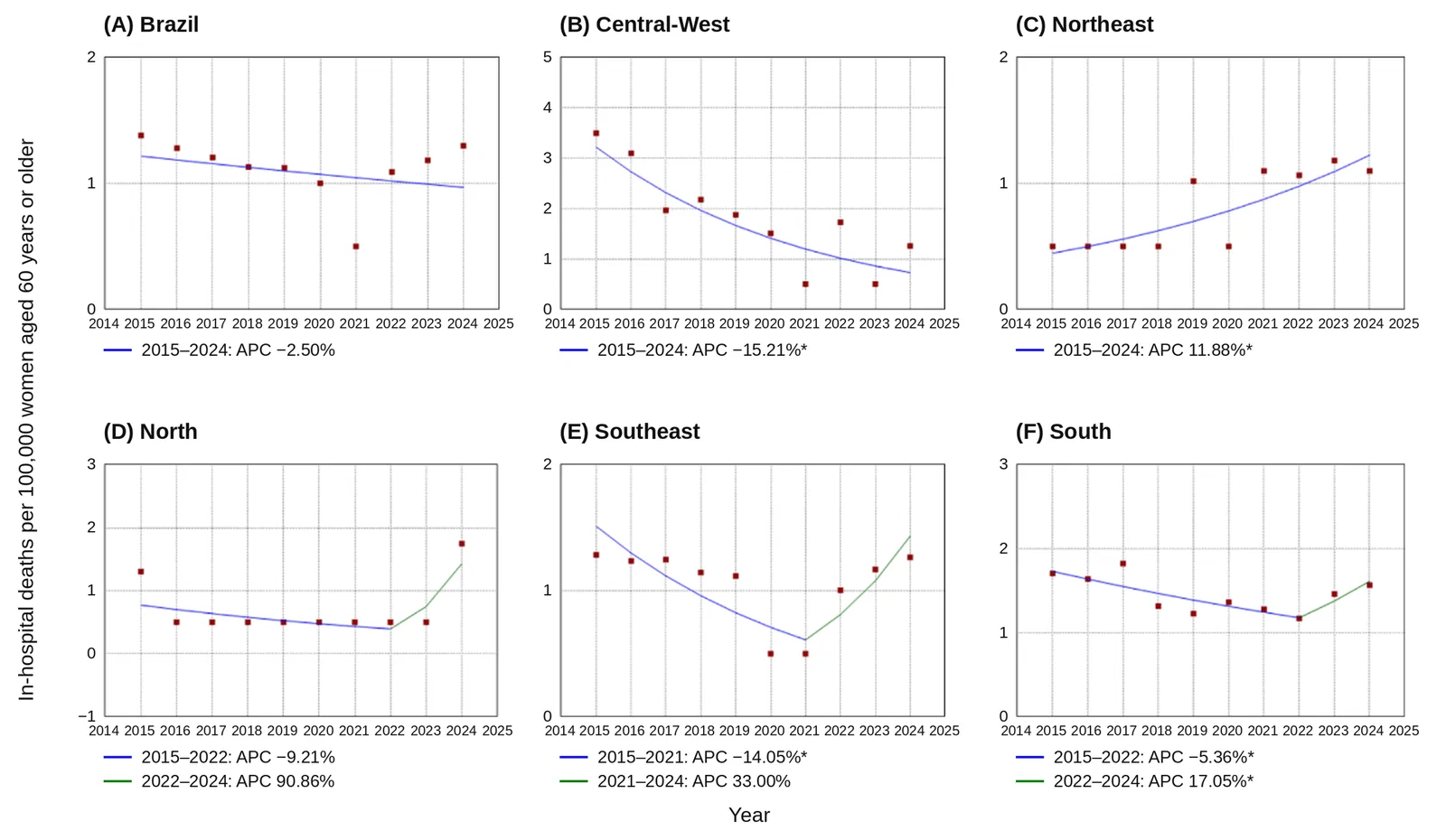
Temporal trends in in-hospital death rates associated with CRHD among women aged 60 years or older in Brazil and its macroregions, 2015–2024. Authors’ analysis of SIH/SUS–DATASUS data using Joinpoint Regression Program v6.0.1 (National Cancer Institute, Bethesda, Maryland). Panels: (A) Brazil; (B) Central-West; (C) Northeast; (D) North; (E) Southeast; (F) South. No joinpoint was selected in (A–C); one was selected in (D–F). *Annual percent change significantly different from zero at the 0.05 level. Blue line, first fitted segment; green line, second fitted segment, where present; red squares, observed annual rates. CRHD: chronic rheumatic heart disease; SIH/SUS: Hospital Information System of the Unified Health System; DATASUS: Department of Informatics of the Unified Health System; APC: annual percent change.

Among men, the Southeast showed a sustained decline throughout 2015-2024 (APC = −9.81%; p = 0.023). In the North, a significant decline during 2015-2018 (APC = −44.55%) was followed by a nonsignificant trend during 2018-2024. The Northeast showed no significant trend during 2015-2021, followed by a significant increase during 2021-2024 (APC = 45.21%). The decline in the Central-West did not reach statistical significance (APC = −8.97%; p = 0.068), and neither segment was statistically significant in the South (Figure 4).

**Figure 4:**
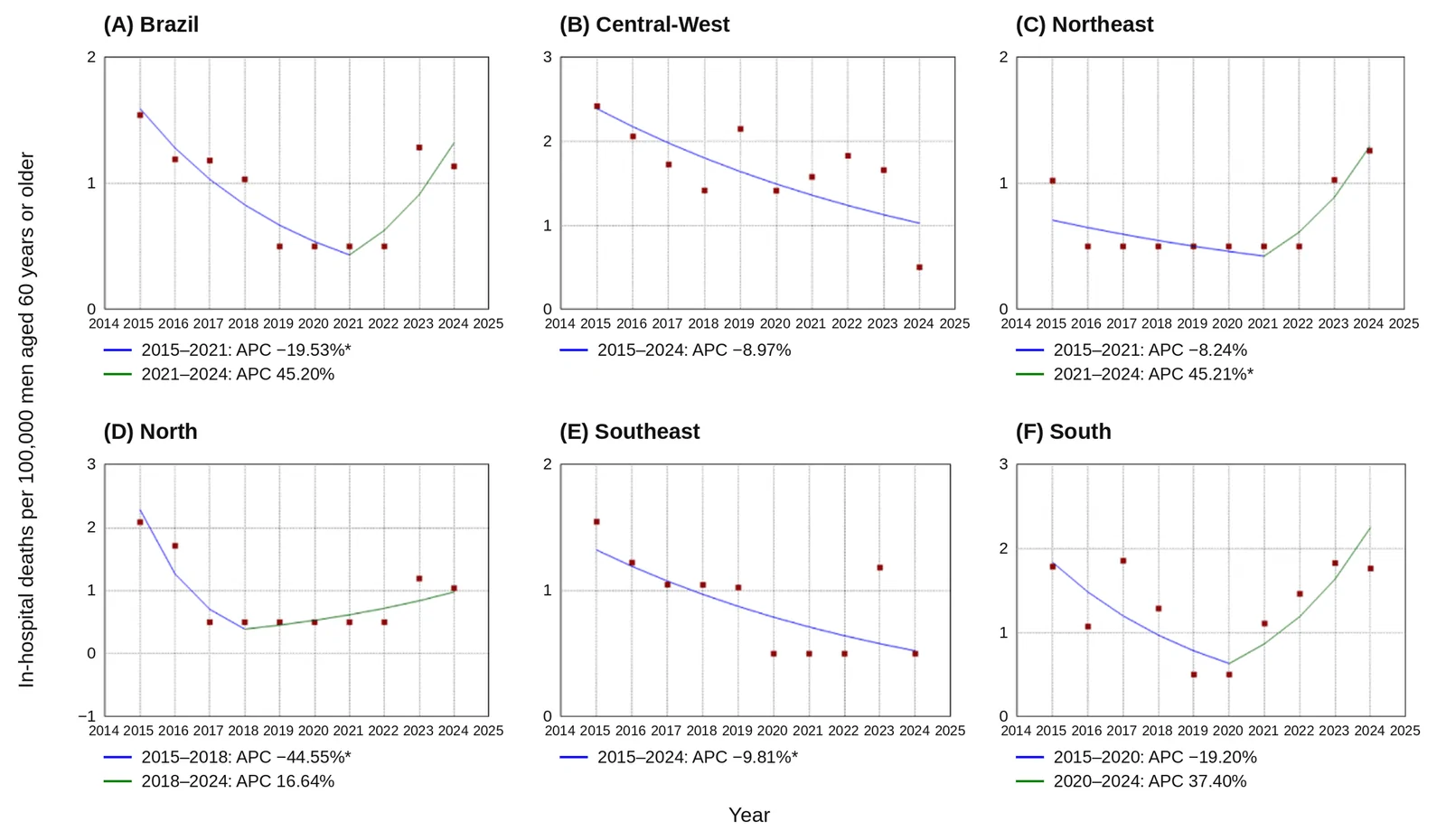
Temporal trends in in-hospital death rates associated with CRHD among men aged 60 years or older in Brazil and its macroregions, 2015–2024. Authors’ analysis of SIH/SUS–DATASUS data using Joinpoint Regression Program v6.0.1 (National Cancer Institute, Bethesda, Maryland). Panels: (A) Brazil; (B) Central-West; (C) Northeast; (D) North; (E) Southeast; (F) South. One joinpoint was selected in (A), (C), (D), and (F); none was selected in (B) or (E). *Annual percent change significantly different from zero at the 0.05 level. Blue line, first fitted segment; green line, second fitted segment, where present; red squares, observed annual rates. CRHD: chronic rheumatic heart disease; SIH/SUS: Hospital Information System of the Unified Health System; DATASUS: Department of Informatics of the Unified Health System.

## Discussion

Hospitalization rates for CRHD among older Brazilian adults declined before rebounding around 2020-2021, while population-based in-hospital death rates followed more heterogeneous trends by sex and region. Neither outcome showed a statistically significant average national trend over the full study period. This summary, however, obscures substantial changes within the decade and the sustained increases among women in the Northeast. The principal contribution of these findings is to characterize how the hospital burden of CRHD has changed in an aging population, while distinguishing the frequency of hospital use from the occurrence of fatal outcomes.

The continued demand for hospital care is consistent with the persistence of rheumatic heart disease worldwide. Analyses of the Global Burden of Disease (GBD) 2021 dataset describe a large population living with the disease despite declining age-standardized mortality, with a disproportionate burden in socioeconomically disadvantaged settings [7,8]. Decomposition of these estimates indicates that population growth and aging can contribute to increasing absolute burden even as standardized indicators improve [8]. In our study, the marked increase in admission counts contrasted with similar crude hospitalization rates at the beginning and end of the period, emphasizing the relevance of demographic change. Hospitalizations in later life may also reflect complications of valvular injury acquired decades earlier [1].

Comparisons with Brazilian studies require attention to the populations, periods, and outcomes examined. National cardiovascular statistics document a long-term decline in age-standardized mortality from rheumatic heart disease [3]. A separate analysis reported declining age-adjusted CRHD mortality during 2000-2021, although its stated use of hospital records limits interpretation as total population mortality [9]. Conversely, an earlier SIH/SUS study described increases in hospital mortality indicators and expenditures associated with rheumatic heart disease [10]. These findings are not directly comparable with crude rates restricted to adults aged 60 years or older. Together, they show why hospitalization rates, deaths during hospitalization per population, and the proportion of admissions ending in death must be interpreted as distinct measures.

The reversal in hospitalization trends around 2020-2021 should be considered in the context of disruptions to cardiovascular care during the COVID-19 pandemic. Brazilian studies documented fewer cardiovascular admissions, including those for rheumatic disease, and reductions in valve surgery and percutaneous mitral valvuloplasty [11,12]. The Latin American INCAPS COVID study also found a sharp decline in cardiac diagnostic procedures [13]. Reduced access to evaluation and treatment could therefore have contributed to the low hospitalization rates observed around this period. The subsequent increase may reflect resumed services and accumulated demand, potentially accompanied by changes in the severity of patients reaching hospital care. However, hospitalization rates had already been falling before 2020, and joinpoint regression cannot isolate the pandemic's effect. The rebound is best interpreted as a change in hospital utilization whose clinical and organizational drivers remain unresolved.

Fatal outcomes remained common among recorded admissions. Although the proportion ending in death was lower in the final year than in the first, this difference alone cannot establish an improvement in care because admission thresholds, case mix, and treatment availability may have changed. Clinical cohorts help identify the factors missing from this administrative perspective. A large international study associated mortality with heart failure, pulmonary hypertension, and atrial fibrillation [14], while the REMEDY registry identified severe valve disease, advanced functional class, and older age as adverse prognostic factors [15]. These cohorts enrolled predominantly younger patients and measured deaths over follow-up. Their mortality estimates therefore provide clinical context rather than a direct benchmark for the proportion of hospitalizations ending in death in our study.

Evidence from valve surgery further illustrates the clinical relevance of age. A nationwide Brazilian cohort found higher in-hospital mortality and poorer subsequent survival among older surgical patients [16]. In a more recent study restricted to patients aged 60 years or older with rheumatic mitral disease, propensity score matching revealed no statistically significant difference in late mortality between valve repair and bioprosthetic replacement [17]. This result does not establish equivalence, but it illustrates the need to consider treatment selection when evaluating outcomes in older adults. Because our records do not distinguish procedural status or surgical indication, the observed hospital burden cannot be attributed to a particular intervention or interpreted as a measure of operative risk.

The sex distribution also differed according to the measure examined. Women accounted for more admissions and in-hospital deaths overall, whereas population-based hospitalization rates were consistently higher among men. The higher unadjusted proportion of admissions ending in death among women likewise requires consideration of age distribution, disease severity, and comorbidities. A greater female disease burden in GBD estimates [7,8] does not necessarily imply worse outcomes after hospitalization. Female sex was associated with lower adjusted mortality in REMEDY [15], and Brazilian surgical data showed different sex patterns for in-hospital mortality and subsequent survival [16]. These differences favor an interpretation grounded in population structure and clinical context. Moreover, differences in statistical significance between sex-specific trends do not establish that the trends themselves differ.

The Northeast warrants particular attention because hospitalization and in-hospital death rates among women increased throughout the study period. The persistence of these trends suggests that the regional pattern extends beyond a temporary disruption during the pandemic. Brazilian GBD modeling identified less favorable historical prevalence trends in the North and Northeast [18], while a national study of acute rheumatic fever found a concentration of admissions in the Northeast [19]. These studies support the broader context of geographic inequality, although they examine different populations and stages of disease. Evidence on care delivery offers a more direct framework for interpreting these differences: a qualitative study in Salvador identified barriers across primary and specialist services that could impede prevention, diagnosis, and continued care [20]. Whether these barriers contributed to the trends observed here requires investigation with individual-level and health service data.

Access to specialized care may also shape regional hospital outcomes. A recent Brazilian cohort linking administrative databases found that income and geography were associated with survival after valve replacement [21]. The BYPASS registry documented the continuing prominence of rheumatic disease among surgical valve patients and the substantial role of SUS financing [22]. Although neither study was restricted to older adults with CRHD, both reinforce the relevance of referral pathways and the distribution of surgical services. In our data, the North combined relatively low hospitalization rates with the highest cumulative proportion of admissions ending in death. This combination warrants investigation of disease severity at admission and access to timely care; the low admission rate alone does not establish a low underlying disease burden.

The national coverage and explicit focus on older adults make these findings relevant to planning hospital and specialist services. They support attention to continuity of care and timely specialist assessment, particularly where hospital burden is increasing. In the international cohort, valve surgery and valvuloplasty were associated with lower mortality, supporting further evaluation of access to intervention while acknowledging confounding and treatment selection [14]. Linking hospital records to outpatient, procedural, and mortality data would help distinguish repeat admissions from first hospitalizations, characterize treatment pathways, and capture outcomes after discharge. Such analyses could clarify whether regional increases reflect improved access, greater unmet clinical need, or both.

Limitations should be considered when interpreting these findings. SIH/SUS captures hospitalizations funded by the Brazilian Unified Health System (SUS) but does not fully include care financed exclusively by the private sector. In addition, records represent hospitalization events rather than unique individuals, meaning that repeated admissions by the same patient may contribute to the observed burden. Therefore, changes in hospitalization rates should not be interpreted directly as changes in the number of affected individuals or disease incidence. Case identification also depends on the accuracy of diagnostic coding in Hospital Admission Authorizations. Furthermore, the aggregated data do not provide information on clinical severity, valvular characteristics, comorbidities, previous treatments, or indications for hospitalization. Deaths were recorded only when they occurred during hospital stays and thus do not reflect total population mortality from CRHD. Comparisons across regions should also account for potential differences in the age composition of the population aged 60 years or older, as well as referral patterns and access to hospital services. The relatively short annual series, the limited number of observations in some joinpoint segments, and the stratification of analyses warrant caution when interpreting temporal trends. Finally, the ecological design limits individual-level inferences and causal interpretations. As discussed above, the changes observed around 2020-2021 should be considered in the context of the COVID-19 pandemic and its disruption of cardiovascular care. Although the subsequent increase in hospitalizations may reflect the resumption of services and accumulated demand, the available data do not allow these contributions to be distinguished from other changes in healthcare utilization.

## Conclusions

Among older Brazilian adults, CRHD hospitalization rates declined and then rebounded around 2020-2021, while population-based in-hospital death rates followed more heterogeneous trends. Average national trends over the full decade obscured changes within shorter periods and sustained increases among women in the Northeast. These findings identify a continuing and geographically uneven burden on SUS-funded hospital care. The recent rise in admissions reflects greater hospital utilization and does not establish an increase in disease incidence. Priorities include continued surveillance, continuity of follow-up, and evaluation of regional access to specialist care. Linked clinical and administrative data are needed to determine the drivers of these patterns and identify opportunities to improve outcomes.
